# Influence of sub-clinical endometritis on early pregnancy predictors and proinflammatory cytokines in circulating immune cells in dairy cows

**DOI:** 10.1590/1984-3143-AR2023-0144

**Published:** 2024-07-15

**Authors:** Diego Angelo Schmidt Poit, Priscila Assis Ferraz, Gabriela de Andrade Bruni, Giulia de Andrade Bruni, Thiago Kan Nishimura, Igor Garcia Motta, Isabella Rio Feltrin, Guilherme Pugliesi

**Affiliations:** 1 Departamento de Reprodução Animal, Faculdade de Medicina Veterinária e Zootecnia, Universidade de São Paulo, Pirassununga, SP, Brasil; 2 Departamento de Produção Animal, Faculdade de Medicina Veterinária e Zootecnia, Universidade Estadual Paulista, Botucatu, SP, Brasil

**Keywords:** immune response, interferon, pregnancy, Th1 cytokines, uterine disease

## Abstract

In Experiment 1, PBMC were isolated from cows considered healthy or with SCE (n=6/group) on Days 0 (estrus) and 7 (diestrus) of a synchronized estrous cycle. In Experiment 2, on D21 (D0 was defined as the day of Fixed Timed Artificial Insemination (FTAI), cows were evaluated by ultrasonography to assess luteal blood perfusion and PBMC were isolated. On D32, cows were classified into: healthy pregnant (n=7), pregnant with SCE (n=4), healthy non-pregnant (n=8), and non-pregnant with SCE (n=10). Gene expression of ISGs (ISG15, OAS1, MX1 and IFI6) and proinflammatory cytokines (IL1-β, TNF-α and IFN-γ) were determined. Expression of ISG15, MX1, IFI6, TNF-α and IFN-γ did not differ between SCE and healthy cows and between Days 0 and 7. Expression of OAS1 and IL1-β were higher (P=0.02) on Day 7 than Day 0, regardlees of the SCE presence. In Exp.2, ISG15 abundance was 2.5-fold greater (P=0.0008), TNF-α was 2.2-fold greater (P=0.05), and IL1-β tended (P=0.06) to be 2.4-fold higher in pregnant than non-pregnant cows. Luteal blood perfusion was greater (P=0.01) in pregnant animals. In conclusion, OAS1 and IL1-β are transcripts upregulated in PBMC at diestrus, regardless of SCE occurrence. Proinflammatory cytokines are not affected by SCE occurrence, but IL1-β and TNF-α are upregulated in pregnant animals on D21 of pregnancy. ISG15 abundance is a good pregnancy predictor, regardless SCE presence.

## Introduction

The modulation of the immune system during early pregnancy is the first step for pregnancy establishment in ruminants (Rocha et al., 2021). Interferon-tau (IFN-t) is a cytokine produced by the trophectoderm, released in the uterus, which stimulates the expression of genes (ISGs) in various tissues (Forde et al., 2011) and in immune blood cells (Han et al., 2006; Pugliesi et al., 2014) in cattle. Conceptus signaling through IFN-t modulates the maternal immune response, favoring the establishment of pregnancy, by decreasing the pro-inflammatory response (Th1) and inducing an anti-inflammatory response (Th2). In this context, Rashid et al. (2018) reported that gene expression of *IL1-β* and *TNF-α* (Th1-cytokines) was significantly lower in mononuclear cells (PBMC) cultured with uterine flush from day 7 pregnant cows compared to non-pregnant cows.

Pregnancy establishment in cattle can be influenced by several factors. Uterine diseases, such as clinical endometritis (CE) and subclinical endometritis (SCE), are frequently caused by bacterial infections, with *Trueperella pyogenes* and *Escherichia coli* being the most common pathogens (Williams et al., 2005; McDougall et al., 2007). Recently, the term purulent vaginal discharge has been commonly used for cows with more than 50% of pus in the vaginal discharge, regardless of the presence of neutrophil infiltrate in the uterus (LeBlanc, 2012). These conditions lead to economic losses due to decrease milk production, as well as treatment costs, which can subsequently impact fertilization and embryo development in dairy cattle (Ribeiro et al., 2016; Carvalho et al., 2019).

Subclinical endometritis (SCE), also referred to as cytological endometritis, is characterized by increased leukocyte infiltration in the uterus, mainly of polymorphonuclear cells (PMN), without the manifestation of clinical symptoms (Sheldon et al., 2006). As well as other uterine diseases, SCE causes harmful effects on reproductive performance in high-production dairy cows (Kasimanickam et al., 2004; Salasel et al., 2010; LeBlanc, 2012). Moreover, SCE can be diagnosed by the determination of PMN proportion in cells collected in the uterine lumen by the cytobrush technique (Ghasemi et al., 2012). The proportion of PMN to classify an animal with SCE ranges between 5 and 18% and is dependent on the phase of the postpartum period (Dubuc et al., 2010; LeBlanc, 2012; Denis-Robichaud and Dubuc, 2015).

The inflammatory process generated by uterine diseases also leads to increased expression of pro-inflammatory cytokines in the blood (Galvão et al., 2012) and uterus, which is associated with embryo implantation failures, leading to pregnancy losses during the first weeks of pregnancy (Galvão et al., 2011; Kasimanickam et al., 2014; Kim et al., 2014; Ribeiro et al., 2016). Despite not having evaluated animals with SCE, Ribeiro et al. (2016), highlighted that those cows with retained placenta and puerperal metritis, lead to a smaller conceptus, which potentially results in a reduced capacity of conceptus to secrete IFN-t compared to those in healthy animals. In contrast, induction of an inflammatory process through intramammary (Campos et al., 2018) or systemic (Fernandes et al., 2019) infusion with lipopolysaccharide resulted in a higher expression of ISGs in the endometrium of cows between 15 and 19 days of pregnancy.

In recent years, new technologies to predict early pregnancy around day 20 after timed artificial insemination (TAI), have been developed, including the quantification of ISGs in immune blood cells (Pohler et al., 2016; Yoshino et al., 2018) and Doppler ultrasonography (Pugliesi et al., 2014). However, the mechanisms involved in the false positive results obtained with both techniques are still unclear. The IFN-t receptor (IFNAR) is non-selective, and is stimulated by any type 1 interferon, such as those produced by several inflammatory processes (Takino et al., 2016). However, to date, there is no evidence that animals presenting an inflammatory uterine disease, such as the SCE, can stimulate the expression of ISGs, inducing a greater proportion of false positive results.

Doppler ultrasonography has been used as a tool to early detect pregnancy status on day 20 after TAI through assessing blood perfusion of the corpus luteum (CL), achieving an accuracy greater than 90% and 75% in beef and dairy cows, respectively (Siqueira et al., 2013; Pugliesi et al., 2014; Melo et al., 2020). Therefore, has been speculated that the lower accuracy of this technique in dairy cattle could be attributed to higher embryonic mortality in this subspecies, associated with a greater occurrence of uterine diseases in the early postpartum period, such as clinical endometritis compared to beef cows.

In the present study, our objectives were: 1) to evaluate if the expression of ISGs and pro-inflammatory cytokines in peripheral blood mononuclear cells (PBMC) is modulated in the presence of SCE in dairy cows, and 2) to compare the accuracy of pregnancy early predictors (ISGs and CL blood perfusion) in animals with or without SCE. Therefore, we tested the following hypotheses: 1) the ISGs expression is modulated either the occurrence of SCE or in different estrous cycle phases; 2) pregnant cows will present a reduced expression of pro-inflammatory cytokines in comparison to non-pregnant cows; and 3) the use of ISGs expression as pregnancy predictor at 21 days of pregnancy is less effective in animals that presented SCE.

## Material and methods

### Animals and experimental design

The experiments were carried out following the Institutional Committee for Ethics in Research of the University of São Paulo (CEUA-FMVZ number: 1202270819).

### Experiment 1

Our objective in this experiment was to evaluate if the expression of ISGs and pro-inflammatory cytokines in peripheral blood mononuclear cells (PBMC) is modulated in the presence of SCE in dairy cows. This study was conducted on a commercial farm, located at Itobi, SP-Brazil, from July/2020 to September/2020. For this, primiparous and multiparous Holstein (*Bos taurus*) cows (n=30), between 30 and 40 days postpartum, in a body condition score from 2.5 to 4, on a scale of 1-5 (1: thin and 5: fat) (Ferguson et al., 1994), and with averaged milk yield of 20.5 ± 0.6 kg/day, was used.

The animals were housed in a compost barn and milked thrice daily and were fed a total mixed ration twice daily that consisted of corn silage as forage, with the addition of a corn and soybean meal-based concentrate. The total mixed ration was formulated to meet or exceed the minimum nutritional requirements for lactating dairy cows (NRC, 2001). All animals were submitted to vaginal discharge evaluation using the Metricheck device, transrectal ultrasonography, and rectal temperature on Day −7 (Day 0 = day of expected estrus), for clinical endometritis diagnosis. The vaginal discharge evaluation using the Metricheck device was performed as described previously by McDougall et al. (2007), with minor modifications, using 5 points scale, where, 0 = no discharge, 1 = clear discharge, 2 = discharge with speck of pus (less than 50% of pus), 3 = mucopurulent discharge (more than 50% of pus), 4 = purulent discharge, and 5 = a fetid, watery red-brown discharge. Clinical endometritis was defined by the presence of a purulent discharge (greater than or equal to 50% of pus, scores 3 or 4) detected by a Metricheck device (Sheldon et al., 2006) and/or the presence of inflammatory signs in the ultrasonography (uterine fluid or hyperechoic endometrial surface), without the presence of systemic signs.

Animals without clinical signs of endometritis were submitted to a pre-synchronization protocol on a random day of the estrous cycle. On Day −7 an intravaginal progesterone (P4)-releasing device (Sincrogest^®^, Ourofino Saúde Animal, Brazil) and an intramuscular single dose of a prostaglandin analog (0.526 mg of sodium cloprostenol; Sincrocio^®^, Ourofino Saúde Animal, Brazil) was administrated. After five days (Day - 2), the intravaginal device was withdrawn, and another dose of prostaglandin was administrated, followed by an injection of estradiol cypionate (1 mg estradiol cypionate, SincroCP^®^, Ourofino Saúde Animal, Brazil). On Day 0 (day of expected estrus), animals were evaluated by transrectal ultrasonography and those with the largest ovarian follicle with > 10 mm in diameter received an intramuscular injection of GnRH analog (10 µg of buserelin acetate; Sincroforte^®^, Ourofino Saúde Animal, Brazil) to induce the ovulation, then, were submitted to Cytobrush technique for diagnose of SCE. Blood samples from the coccygeal vessels for gene expression were collected on days Day 0 (estrus) and Day 7 (diestrus).

Transrectal ultrasonography exams were performed to confirm the presence of a dominant follicle and CL, respectively, on Days 0 and 7. Only cows considered clinically healthy, and with the presence of an ovarian dominant follicle (follicle with >10 mm in diameter in the absence of a CL) on Day 0 and the presence of CL on Day 7 were included. Three animals with clinical endometritis, one with a follicular cyst, and four that had no CL presence on Day 7 were identified and excluded for the subsequent procedures. On Day 0, the remaining 22 Holstein dairy cows were classified based on the PMN counting in the cytological samples collected by the Cytobrush technique, according to Madoz et al. (2013). For classification of SCE occurrence, only cows with ≥ 5.5% of PMN were considered with SCE (SCE group; n=6), and those with ≤ 3.0% of PMN were considered without any uterine disease (NUD group; n=6).

### Experiment 2

In this study, our objective was to compare the accuracy of early pregnancy predictors (ISGs and CL blood perfusion) in animals with or without SCE. The study was carried out at a commercial farm, located in Arceburgo, MG- Brazil, from January 2021 to April 2021. At the enrollment, 50 Holstein *(Bos taurus)* dairy cows, primiparous and multiparous, between 40- and 55 days post-partum, in a body condition score from 2.5 to 4, and with average milk yield of 24.3 ± 0.8 kg/day, which would be submitted to first service, were used. Cows were housed in a compost barn and milked thrice daily and were fed a total mixed ration twice daily that consisted of corn silage and alfalfa silage as forage, with the addition of a corn and soybean meal-based concentrate. The total mixed ration was formulated to meet or exceed the minimum nutritional requirements for lactating dairy cows (NRC, 2001).

Cows were submitted to vaginal discharge evaluation using the Metricheck device for clinical endometritis diagnosis and those (n=48) without any signal of clinical uterine diseases were submitted to a hormonal protocol for TAI. The Metricheck device was performed as described in the *Experiment 1*. Two cows were excluded from the study because they had a vaginal discharge score ≥ 3. The day of TAI was considered as Day 0 (D0) of the protocol for synchronization of ovulation, which started 10 days before (D−10). On Day -10, an intravaginal P4-releasing device (Fertilcare 1200®, MSD, Brazil) was inserted, followed by an intramuscular injection of estradiol benzoate (2 mg; Sincrodiol®, Ourofino Saúde Animal, Brazil) and an analog of GnRH (10 µg of gonadorelin acetate; Fertagyl®, MSD, Brazil). On D-3 and D-2, a prostaglandin analog (0.526 mg of sodium cloprostenol; Sincrocio®, Ourofino Saúde Animal, Brazil) was administered 24 hours apart. Still on D-2, the intravaginal device was removed, followed by an injection of estradiol cypionate (1 mg; SincroCP®, Ourofino Saúde Animal, Brazil). On D0 (TAI day), an intramuscular injection of a GnRH analog (10 µg of gonadorelin acetate; Fertagyl®, MSD, Brazil) was administered to induce ovulation and then the animals were inseminated. Inseminations were performed by a single technician using semen from one of four sires.

Considering that the insertion of cytological brush in the uterine lumen after the insemination could disturb the establishment of pregnancy, the SCE presence was diagnosed using the Cytobrush technique at the beginning of the TAI protocol (D−10). For the classification of SCE occurrence, considering that cows were at a later postpartum period than in *Experiment 1*, only cows with ≥ 5% of PMN were considered with SCE (n=14), as previously described by Madoz et al. (2013). Females with ≤ 2.0% PMN (n=15) were considered without uterine disease. Doppler ultrasonography and blood samples collection for gene expression were performed on D21 after TAI. The final pregnancy diagnosis was performed on D32, under the presence of a viable embryo with heartbeats. Therefore, after pregnancy diagnosis, the animals were allocated in four experimental groups: NUD and pregnant (NUD-P, n=7); NUD and non-pregnant (NUD-NP, n=8); SCE and pregnant (SCE-P, n=4); and SCE and non-pregnant (SCE-NP, n=10).

### Cytobrush technique

Cytobrush was performed, for both experiments as previously detailed by Cardoso et al. (2017). The content present in the brush was passed to a microscope slide (26x76x1 mm), by a careful rolling of the brush over the surface of the slide, and then, the slides were stained by the rapid Panotic staining kit (Laborclin, Pinhais, Brazil) for visualization in the optical microscope. For the determination of SCE, the proportion of PMN in the sample was determined based on counting of approximately 200 cells.

### Ultrasound scanning

In *Experiment 1*, the uterine illness evaluation and ovary structures detection (CL or dominant follicle), were performed using a B-mode instrument (DP-50 Vet, Mindray, China) with a linear multi-frequency (3.5-7.5 MHz) transducer in B-mode (gain 71%, frequency 7.5 MHz, P 74 mm, IP 4, depth 55 mm).

In *Experiment 2*, the cows were evaluated by transrectal ultrasonography using a duplex ultrasound equipment (ExaPad mini, IMV Imaging, USA) with a linear multi-frequency (3.5-7.5 MHz) transducer in B-mode (gain 70%, depth 60 mm, Dynamic Range 70 dB) and color Doppler mode (gain 61%, PRF 1 kHz, frequency 7.1 MHz, WF 2). The examination of the luteal area with color Doppler signals of blood perfusion (%) at each exam was determined as previously described by Rocha et al. (2019). Cutoff point (luteal blood perfusion ≥ 25%) for the Doppler ultrasonography method (Doppler-US) was used to identify the females with a functional CL and they were classified as pregnant.

### Isolation of Peripheral Blood Mononuclear Cells (PBMC)

Blood samples collected for PBMC (≈30 mL) were submitted to a Ficoll gradient protocol, as described by Pugliesi et al. (2014). Resulting PBMC pellet was stored in a 1.5 mL conical tube at −80 °C until RNA extraction. To check the purity, freshly isolated samples were placed on a slice and stained with fast panotic method for morphological identification of cells by light microscopy under 400 × magnification. The purity >95% for all samples.

### RNA extraction, cDNA synthesis and qPCR

Isolated PBMC were submitted to RNA extraction using the Trizol^®^ Kit (Invitrogen, Paisley, UK) as recommended by the manufacturer, according to the manufacturer's instructions.

For the synthesis of cDNA, firstly, the gene DNA was removed using DNase and the TURBO DNA-free kit (Ambion, Austin, TX, USA), and a microgram of total RNA will be transcribed into cDNA using random primers and a reverse transcription kit High Capacity (Applied Biosystems, Foster City, CA, USA). qPCR quantification was performed using the Power Mix SYBR Green PCR Master Mix (Thermo Fisher Scientific) and the ABI7300 real-time PCR system (Applied Biosystems). Two reference genes with the most stable expression were used for this specific cell type, *GAPDH* and *PPIA*. The expression of the target genes (*ISG15*, *OAS1*, *MX1*, *IFI6*, TNF-α, *IFN-γ, IL-1β* and *IL-10*), were carried out using the comparative method (ΔΔ) CT as described by Pfaffl (2001), being normalized in relation to the two reference genes mentioned above. The following primers of *ISG15, OAS1* and *MX1* were taken from Pugliesi et al. (2014); *IFI6* from Rocha et al. (2020); *IL1-β* and *IFN-γ* from Talukder et al. (2018), *TFN-α* from Rashid et al. (2018) and *IL-10* from Talukder et al. (2017) (Table 1). The primers of housekeeping genes, were previously described by Dalmaso de Melo et al. (2020) and Rocha et al. (2020) (Table 1).

**Table 1:** Detail of primers used in Experiments 1 and 2. Forward (F) and reverse (R) primers sequences of target and reference genes analyzed using qPCR.

**Target**	**Gene Bank Number**	**Forward primer sequence (5'-3')**	**Reverse primer sequence (5'-3')**
***ISG15***	NM_174366	GGTATCCGAGCTGAAGCAGTT	ACCTCCCTGCTGTCAAGGT
***OAS1***	NM_001040606.1	TAGCCTGGAACATCAGGTC	TTTGGTCTGGCTGGATTACC
***MX1***	NM_173940.2	GTACGAGCCGAGTTCTCCAA	ATGTCCACAGCAGGCTCTTC
***IFI6***	NM_001075588.1	TGCTCTCCTCCAAGATACGGT	CAGAAGCTCGAGTCGCTGTT
***IL1-β***	NM_174093.1	AATCGAAGAAAGGCCCGTCT	ATATCCTGGCCACCTCGAAA
***IFN-γ***	NM_174086	AGCTCTGAGAAACTGGAGGACT	TGGCTTTGCGCTGGATCT
***TFN-α***	NM_173966.3	CAAAAGCATGATCCGGGATG	TTCTCGGAGAGCACCTCCTC
***IL10***	NM_174088.1	GAGATGCGAGCACCCTGTCT	GGCTGGTTGGCAAGTGGATA
***GAPDH***	NM_01034034.2	GCCATCAATGACCCCTTCAT	TGCCGTGGGTGGAATCA
***PPIA***	BF230516.1	GCCATGGAGCGCTTTGG	CCACAGTCAGCAATGGTGATCT

### Statistical analyses

The statistical analysis of the gene expression was performed using ANOVA, considering the random effect of the cow and the fixed group effects (with or without SCE). For *Experiment 1*, the analysis was performed considering the gene expression as repeated measurement (Day 0 and Day 7), and the interaction between them (SCE x day of sampling). For the *Experiment 2*, as 2x2 factorial, considering the effects of disease or pregnancy status and its possible interaction, using the PROC mixed software of the SAS (Version 9.2; SAS Institute). The LSD was used for comparisons among more than two means. The variables were tested for normality of residues and homogeneity of variances and when it did not occur the data were transformed to Log or ranked. In *Experiment 2*, a ROC curve was performed to determine the better cutoff of % of PMN for SCE presence, based on the pregnancy per AI. The accuracy of the pregnancy diagnosis methods by ISGs and Doppler ultrasonography on D21 post-TAI and comparison with B-mode ultrasound method on D32 (Gold Standard) was calculated by specificity and sensitivity, as previously described by Pugliesi et al. (2014). Pearson’s correlation among PMN proportion and gene expression of ISGs and pro-inflammatory cytokines was analyzed by PROC Corr of SAS. A cutoff value for ISGs expression and luteal blood perfusion was determined through establishment of a Receiver operating characteristic (ROC) curve. The MedCalc software was used for creation of the ROC curves and to determine the area under the curve (AUC) of each method. Were considered as significant difference when *P ≤ 0.05*, and as trend when it is between *0.05 < P < 0.10*. Data were expressed as mean ± standard error.

## Results

### Experiment 1

The PMN proportion (%) of cytological samples in the NUD and SCE groups ranged, respectively, from 1 to 3 (mean: 2.1 ± 0.3) and 5.5 to 27.5 (mean: 9.7 ± 3.6). The mean ± SEM days of cytobrush and body condition score in *Experiment 1*, were, 40.08 ± 0.6 and 2.96 ± 0.1, respectively.

**Figure 1:**
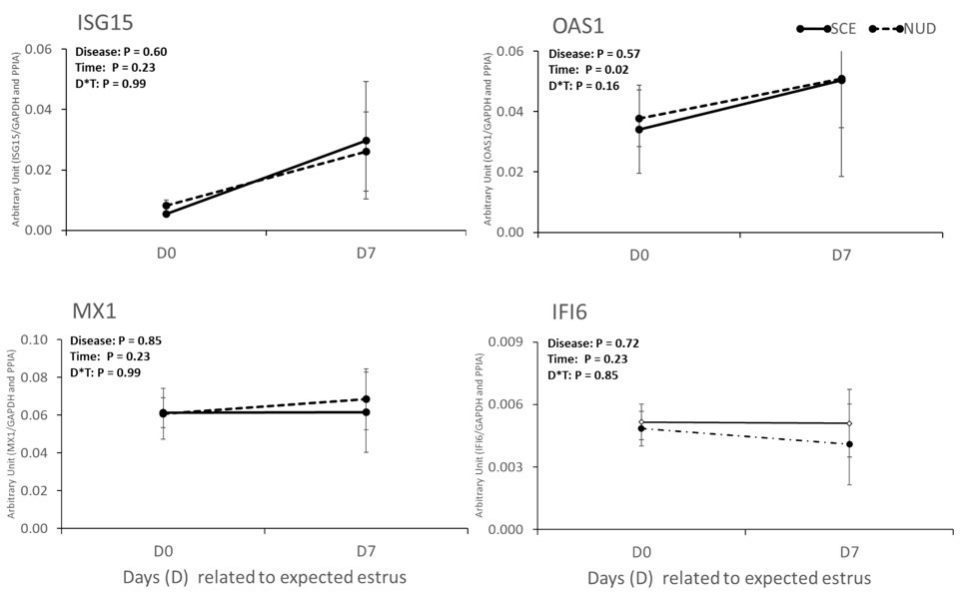
Relative expression of ISGs (*ISG15*, OAS1, *MX1*, *IFI6*) in PBMC on Days 0 and 7 of dairy cows with subclinical endometritis (SCE; n=6) or non-uterine disease (NUD; n=6). The main effects of disease (D), time (T) and their interaction (D*T) were considered significant difference when, P < 0.05.

The expression of the four ISGs evaluated is shown in Figure 1. The abundance of *ISG15*, *MX1*, and *IFI6* was not associated with the occurrence of SCE, time of sampling, or an interaction of SCE and time of evaluation (*P > 0.1*). However, the abundance of *OAS1* transcript was 1.4-fold higher on Day 7 compared to Day 0 (*P = 0.02*).

**Figure 2:**
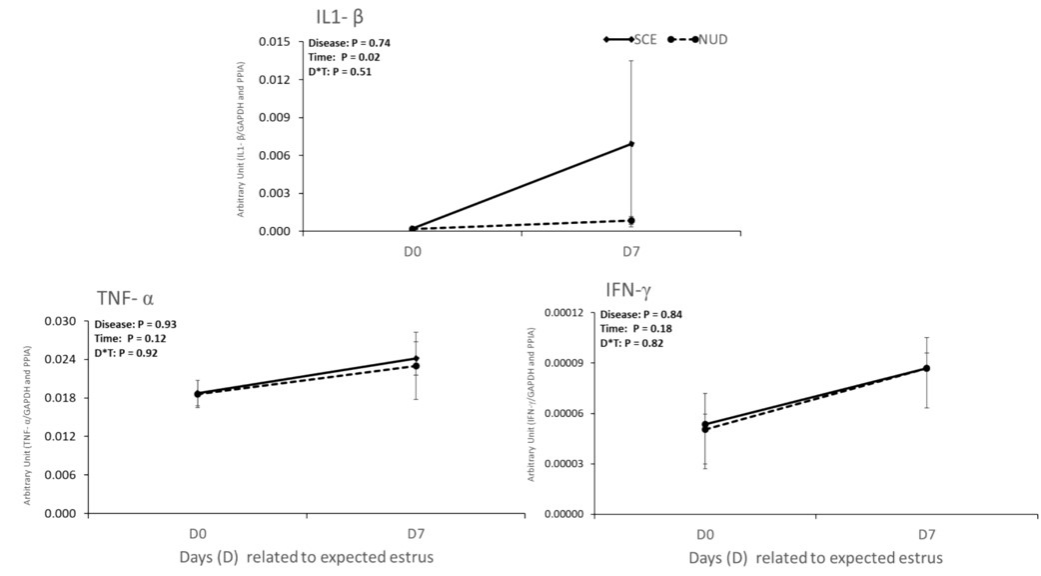
Relative expression of cytokines (*IL1-β, TNF-α* and *IFN-γ*) in PBMC on Days 0 and 7 of dairy cows with subclinical endometritis (SCE; n=6) and non-uterine disease (NUD; n=6). The main effects of disease (D), time (T) and their interaction (D*T) were considered significant difference when, P < 0.05.

Gene expression of the three proinflammatory cytokines is shown in Figure 2. The abundance of *TNF*-α and *IFN-γ* was not different between animals with disease (NUD or SCE) or an interaction of SCE and time of sampling was observed. However, for *IL1-β,* the abundance of *IL1-β* was 19.3-fold higher on Day 7 compared to Day 0 (*P = 0.02*).

### Experiment 2

Pregnancy per AI (P/AI) in SCE and NUD, was respectively, 28.6% (4/14) and 46.7% (7/15).

#### Proportion of PMN at the beginning of the TAI protocol

The PMN proportion (%) of cytological samples ranged from 1 to 2 in the NUD group and from 5 to 17 in the SCE group. The averaged mean of PMN proportion (%) of cytological samples for NUD-P, NUD-NP, SCE-P, and SCE-NP were, respectively, 1.7 ± 0.2; 1.5 ± 0.2; 6.2 ± 0.8 and 8.2 ± 1.1. The proportion of PMN did not differ (*P > 0.1*) between pregnant and non-pregnant animals in NUD and SCE groups. The mean ± SEM days of cytobrush and body condition score in *Experiment 2*, were, 44.97 ± 0.6 and 2.78 ± 0.1, respectively.

#### Gene expression in PBMC on D21 after TAI

**Figure 3:**
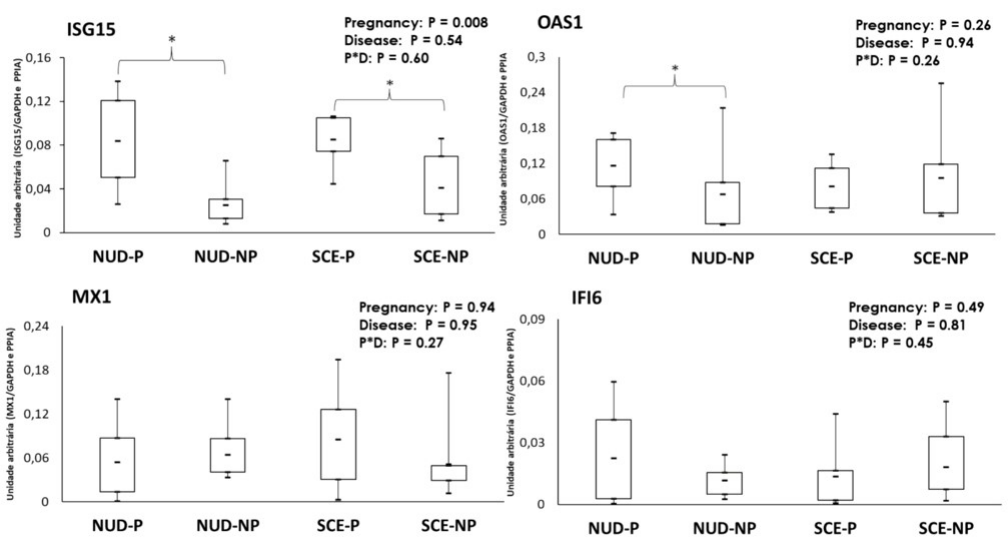
Box plots for the abundance of ISGs (*ISG15*, *OAS1*, *MX1* and *IFI6*) in PBMC on D21 post-TAI from non-uterine disease and pregnant (NUD-P; n=7); non-uterine disease and non-pregnant (NUD-NP; n=8), subclinical endometritis and pregnant (SCE-P; n=4), sub-clinical endometritis and non-pregnant (SCE-NP; n=10) dairy cows. The boxes show the interquartile range, means are indicated by continuous midlines, whiskers are showing the maximum and minimum value in each group. The main effects of disease (D) and pregnancy (P) that were significant are shown with an asterisk (*), which indicates statistical difference between the means among the groups (P < 0.05).

While the *ISG15* abundance was 2.47-fold greater (*P < 0.001*) for pregnant cows, regardless of uterine status (NUD or SCE), the abundance of the other ISGs (*OAS1*, *MX1,* and *IFI6*) was not different between the pregnancy statuses nor uterine statuses (Figure 3). Although a significant interaction of pregnancy status by SCE presence was not detected on gene expression for any ISGs when the pregnant and non-pregnant cows were compared separately in each uterine status (NUD or SCE), the *OAS1* expression in pregnant cows from the NUD class was 1.72-fold greater (*P < 0.1*) than non-pregnant cows, but did not differ (*P > 0.1*) between pregnancy status in the SCE class.

**Figure 4:**
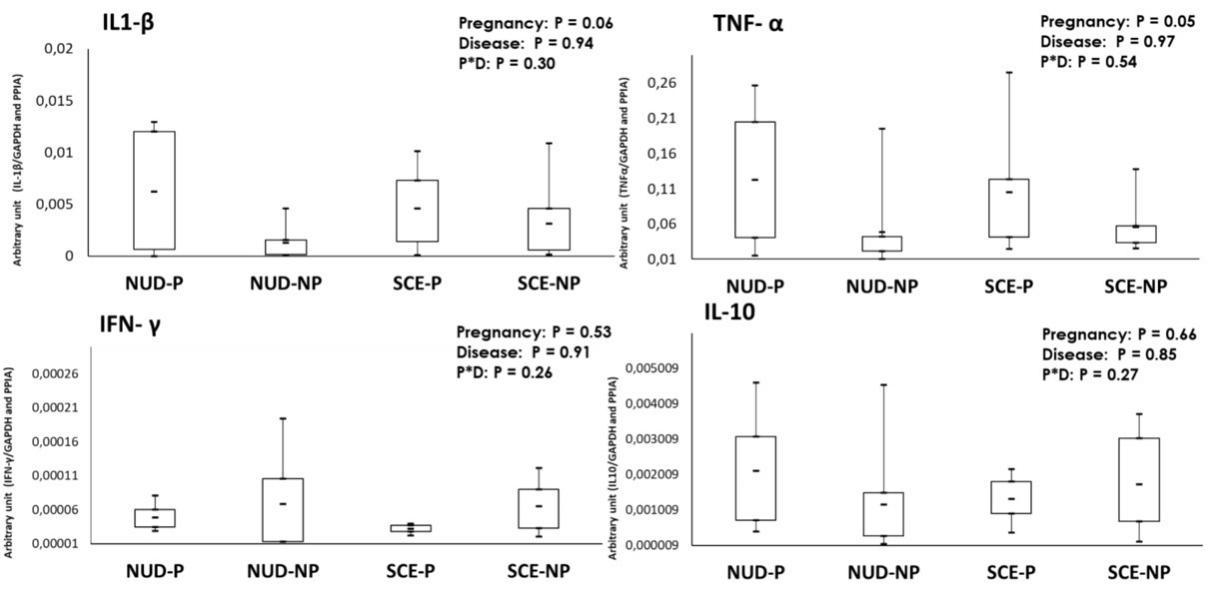
Box plots for the abundance of proinflammatory cytokines (*IL1*-*β*, *TNF*-*α* and *IFN*-*γ) and* anti-inflammatory cytokine *(IL-10*) in PBMC on D21 post-TAI from non-uterine disease and pregnant (NUD-P; n=7); non-uterine disease and non-pregnant (NUD-NP; n=8), subclinical endometritis and pregnant (SCE-P; n=4), sub-clinical endometritis and non-pregnant (SCE-NP; n=10). The boxes show the interquartile range, means are indicated by continuous midlines, whiskers are showing the maximum and minimum value in each group. The main effects of disease (D) and pregnancy (P) that were significant are shown with an asterisk (*), which indicates statistical difference between the means among the groups (P < 0.05).

Gene expression of cytokines is shown in Figure 4. A significant effect of SCE presence on gene expression was not detected for any cytokine (*P > 0.1*). The abundance of *IFN-γ* and *IL-10* did not differ between the uterine or pregnancy statuses, nor an interaction of pregnancy status by SCE presence was observed (*P > 0.1*). Nevertheless, gene expression of *IL1-β* tended to be greater (2.39 - fold) in pregnant than non-pregnant cows, regardless of the SCE presence; whereas, the abundance of *TNF-α* was significantly greater in pregnant than non-pregnant (2.21- fold).

#### Correlations between gene expression and PMN

When Pearson correlations were determined among ISGs and cytokines (Table 2), fifteen significant (*P < 0.05*) and two tendency (*P < 0.1*) positive correlations were detected in the variables evaluated on D21 post-TAI. Among them, 8 of 18, were weak (r < 0.6), five were moderate (0.6 < r < 0.8); whereas three strong correlations (r > 0.8) were observed. There was no significant correlation detected between PMN proportion with any target gene evaluated. Between the ISGs, significant correlations were observed for *OAS1* vs *ISG15*, *IFI6* vs *ISG15*, *IFI6* vs *OAS1,* and *IFI6* vs *MX1*. Significant correlations were observed among all cytokines. Regarding the correlations between the ISGs and cytokines, a significant correlation was detected for *ISG15* with *IL1*-*β*, *TNF-α,* and *IL-10,* and for *OAS1* with *IL1*-*β*, *TNF-α* and *IL-10*.

**Table 2:** Pearson’s correlation coefficient (r) among the PMN proportion in the uterine cytological sample at the beginning of FTAI protocol and abundance of transcripts in PBMC on 21 days post-FTAI in dairy cows.

**Endpoint**	**Gene *vs* PMN**		**Endpoint**	**Between ISGs or Cytokines**		**Endpoint**	**ISGs *vs* Cytokines**	
	**r**	**P**		**r**	**P**		**r**	**P**
*ISG15* by PMN	0.02	NS	*ISG15* by OAS1	**0.65**	***0.001***	*ISG15* by *IL1- β*	**0.48**	***0.01***
*OAS1* by PMN	0.03	NS	*ISG15* by *MX1*	0.26	NS	*ISG15* by *TNF-α*	**0.56**	***0.002***
*MX1* by PMN	-0.12	NS	*ISG15* by *IFI6*	**0.40**	***0.03***	*ISG15* by *IFN-γ*	0.14	NS
*IFI6* by PMN	0.05	NS	*OAS1* by *MX1*	0.25	NS	*ISG15* by *IL-10*	**0.55**	***0.003***
*IL1- β* by PMN	0.22	NS	*OAS1* by *IFI6*	**0.39**	***0.04***	*OAS1* by *IL1- β*	**0.45**	***0.01***
*TNF-α* by PMN	0.17	NS	*MX1* by *IFI6*	**0.74**	***<0.0001***	*OAS1* by *TNF-α*	**0.65**	***0.0001***
*IFN-γ* by PMN	0.09	NS	*TNF-α* by *IL1- β*	**0.82**	***<0.0001***	*OAS1* by *IFN-γ*	**0.32**	***0.09***
*IL-10* by PMN	0.15	NS	*IFN-γ* by *IL1- β*	**0.35**	***0.07***	*OAS1* by *IL-10*	**0.56**	***0.02***
			*IFN-γ* by *TNF-α*	**0.55**	***0.002***	*MX1* by *IL1- β*	-0.18	NS
			*IL-10* by *IL1- β*	**0.85**	***<0.0001***	*MX1* by *TNF-α*	-0.24	NS
			*IL-10* by *TNF-α*	**0.86**	***<0.0001***	*MX1* by *IFN-γ*	-0.32	NS
			*IL-10* by *IFN-γ*	**0.55**	***0.003***	*MX1* by *IL-10*	- 0.19	NS
						*IFI6* by *IL1- β*	-0.17	NS
						*IFI6* by *TNF-α*	-0.05	NS
						*IFI6* by *IFN-γ*	0.17	NS
						*IFI6* by *IL-10*	0.05	NS

NS: Non-significant (P>0.1). P<0.05 indicates a significant differences between the correlations. Pearson's correlation coefficient (r): r < 0.6 – weak correlation, 0.6 < r < 0.8 – moderate correlation, r > 0.8 – strong correlation.

#### Corpus luteum (CL) blood perfusion

As expected, the pregnancy status influenced CL blood perfusion. The CL blood perfusion on D21 post-TAI was greater (*P = 0.01*) in pregnant than non-pregnant cows (56.4% *vs.* 31.4%), regardless of the SCE presence.

#### Accuracy of pregnancy predictors

**Figure 5:**
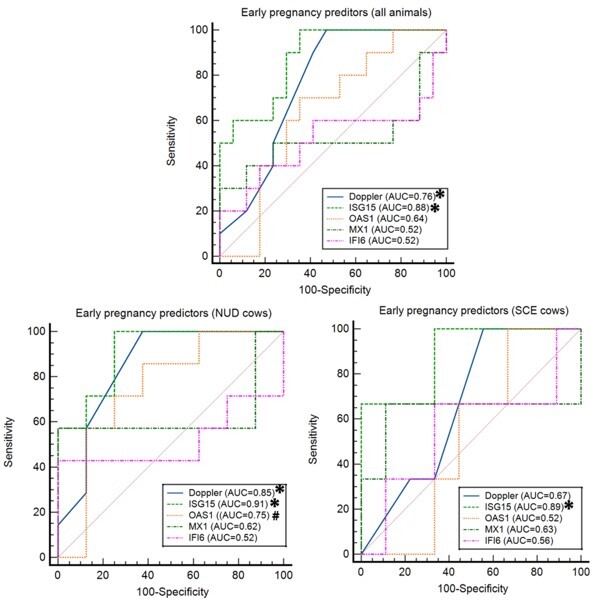
Receiver operator characteristics (ROC) curves for Doppler-US and ISGs on D21 post-TAI for dairy cows (n=29) compared to the diagnosis on D32 post-TAI. An asterisk (*) indicates a significance (P < 0.05) and Hash mark (#) indicates tendency (0.05 ≥ P ≥ 0.10) of the evaluated methods as a pregnancy predictor on D21.

According to the ROC curve analysis (Figure 5), when the gene expression of ISGs was used for the prediction of pregnancy on D32 post-TAI, only the abundance of *ISG15* was detected as a significant predictor (*P < 0.05).* For the exploration of the influence of SCE presence on the accuracy of using ISGs as pregnancy predictors in dairy cows, ROC curves were also generated for NUD or SCE cows apart (Figure 5). Therefore, when analyzed separately (Table 3), the *MX1* and *IFI6* resulted in low sensibility and were still not significant predictors of pregnancy (*P > 0.1*) in SCE and NUD cows; whereas *ISG15* was still considered a significant pregnancy predictor (*P < 0. 0001*) in NUD or SCE cows. However, the specificity of *OAS1* increased when analyzed for NUD cows separately (Table 3), which resulted in a tendency (*P = 0.07*) method of prediction of pregnancy in NUD cows, but not significant in SCE cows. Also, the evaluation of CL blood perfusion on D21 post-TAI was also considered a good predictor, regardless of the SCE presence. However, when analyzed separately of NUD or SCE cows, the sensibility and specificity for CL blood perfusion, by Doppler ultrasonography method were reduced in SCE cows (Table 3), resulting in a significant predictor of pregnancy only in NUD animals.

**Table 3:** Sensitivity (SENS) and Specificity (SPEC), for determining pregnancy status on D21 post-TAI by Doppler-US, *ISG15*, *OAS1, MX1* and *IFI6* gene expression in PBMC compared to diagnosis on D32 post-TAI in dairy cows.

**Target**	**All animals (n=29)**			**NUD cows (n=15)**			**SCE cows (n=14)**		
	**SENS (%)**	**SPEC (%)**	**P Value**	**SENS (%)**	**SPEC (%)**	**P Value**	**SENS (%)**	**SPEC (%)**	**P Value**
**Doppler**	100	52.9	**0.01**	100.0	62.5	**0.001**	80.0	44.4	NS
***ISG15***	100	61.0	**<0.0001**	100.0	75.0	**<0.001**	66.7	100	**0.01**
***OAS1***	72.7	61.1	NS	85.7	62.5	**0.07**	100.0	30.0	NS
***MX1***	30.0	100	NS	57.1	100.0	NS	66.7	80.0	NS
***IFI6***	45.5	100	NS	42.9	100.0	NS	75.0	70.0	NS

## Discussion

The understanding of the maternal immune response to conceptus and uterine diseases is crucial for the development of accurate methods, which can diagnose early pregnancy or pregnancy failures in dairy farms (Fricke et al., 2016). Yet, the factors that compromises the accuracy of ISGs as novel pregnancy biomarkers are still unclear. In the present study, our objectives were to evaluate if the SCE presence is associated with changes in early pregnancy predictors and modulation of pro-inflammatory cytokines gene expression in PBMC. The herein results indicate, for the first time, that the SCE presence at the postpartum period in dairy cattle is not related to the abundance of transcripts for ISGs and pro-inflammatory cytokines in PBMC, but *OAS1* and *IL1-β* are modulated during diestrus of dairy cows, regardless of SCE occurrence. Also, at day 21 of pregnancy, most of the present pro-inflammatory cytokines evaluated are upregulated by the presence of conceptus.

Our first hypothesis that the expression of ISGs is modulated in the presence of SCE or by the estrous cycle phase, even in the absence of the conceptus was not wholly supported. The abundance of ISGs (*ISG15*, *OAS1*, *MX1,* and *IFI6*) in PBMC during the estrous cycle (estrus or diestrus) or at day 21 of pregnancy was not different between cows with or without SCE. Results from non-inseminated cows (*Experiment 1*) show that, among the ISGs only the abundance of *OAS1* was affected by the time of sampling, where, animals had a greater gene expression (1.4-fold) under diestrus than the estrous phase. The *OAS1* gene is involved in the arachidonic acid metabolism, altering the PGF_2α_ secretion in the endometrial epithelium, which prevents the luteolysis (Schmitt et al., 1993). Therefore, the upregulation of the *OAS1* gene in cows under high circulating P4 seven days after expected estrus could be involved in the maintenance of CL function to achieve a successful pregnancy. In addition, the abundance of *IL1-β* was also upregulated at the diestrous phase. In contrast with our results, Talukder et al. (2018), reported in circulating PMN, that *IL1-β* expression did not differ between day 1 (after expected estrus) and day 7 in non-inseminated cows. Therefore, the upregulation in *IL1-β* expression in circulating immune cells may be stimulated by circulating P4, but only after it reaches the maximal concentrations during the estrous cycle (after 10 days post estrus). In *Experiment 1*, we aimed to evaluate the effect of SCE without conceptus or semen interferences, however, further researches are needed to understand, if the semen or conceptus would interfere with gene expression of ISGs, in NUD or SCE cows, during estrous the cycle phase.

Expression of ISGs in PBMC and CL blood perfusion by Doppler ultrasonography, have been used to predict pregnancy in dairy and beef cows around day 20 of pregnancy (Pugliesi et al., 2014; Dalmaso de Melo et al., 2020). In the present study, we evaluated the influence of SCE presence on early pregnancy predictors on D21 after TAI in dairy cows submitted to the first service after calving. The novel results pointed out that only *ISG15* expression on PBMC and CL blood perfusion were different between pregnant and non-pregnant cows, regardless of the SCE presence. That is, pregnant cows had *ISG15* abundance about 2.5 times greater and CL blood perfusion about 1.8 times greater than non-pregnant cows. Similarly, Ribeiro et al., (2016), reported that pregnant cows with no uterine disease diagnosed before insemination had an abundance of *ISG15* in the endometrium, on D19 after insemination, 2.4-fold greater than open cows. However, when evaluating cows diagnosed with retained placenta or clinical metritis before AI, no difference in gene expression of *ISG15* was detected between pregnant and non-pregnant cows. These authors suggest that the capacity of the embryo of those cows to secrete IFN-t, was affected by the uterine disease. A major difference in the previous reports with the present study that may explain the inconsistency in the results is that SCE is a uterine disease with lesser inflammatory response and uterine injuries compared to retained placenta or clinical endometritis.

Previous reports in beef cattle (Green et al., 2010; Pugliesi et at., 2014; Yoshino et al., 2018; Rocha et al., 2020), indicate that gene expression of *IFI6* and *MX1* in PBMC, is greater in pregnant animals when compared to non-pregnant on day 20 after TAI. Those results are not in agreement with the present study, as the abundance of *IFI6* and *MX1* was not affected by the pregnancy status. Although the expression of *MX1* was not correlated with *ISG15* or *OAS1*, *MX1* had a high positive correlation with *IFI6* (r = 0.74). The *IFI6* was also positively correlated with the other two ISGs evaluated on D21 post-TAI. Therefore, these novel results pointed out that *IFI6* and *MX1* expression are correlated but apparently, the conceptus stimulus on expression of both genes is attenuated in dairy cattle. Differences of breed, parity order, and day of sampling between the previous studies performed in *Bos indicus* beef cattle and the present study have also to be considered for interpretation of the stimulus of conceptus presence on expression of *MX1* and *IFI6,* however, further studies are indicated to understand the pregnancy stimulus on those targets in *Bos taurus* dairy cows.

The SCE-induced local inflammation results in an increased expression of pro-inflammatory cytokines in the uterus and blood (Galvão et al., 2011, 2012; Kasimanickam et al., 2014; Kim et al., 2014). Also, these cytokines lead to increased pregnancy losses during maternal recognition of pregnancy (Kasimanickam et al., 2014). The novel results of the present study describe that SCE has no association with the expression of pro-inflammatory cytokines (*IL1-β*, *TNF-α* or *IFN-γ*) in PBMC during the estrous cycle or early pregnancy establishment. However, the abundance of *IL1-β* was affected by the time of sampling, as its expression was 19.3-fold greater on diestrus compared to estrus. Contrary to the present results, Galvão et al. (2011), reported an increase in *IL1-β* expression in the endometrium tissue of cows with PMN ≥ 10% in uterine cytological samples on weeks 5 and 7 of postpartum. In this regard, the inflammatory process associated with SCE is mainly local, and could not modulate the circulating immune cells. However, Fischer et al. (2010) and Kasimanickam et al. (2014) reported, in cows with SCE, a greater gene expression of *IL1-α*, *IL1-β*, and *TNF-α* in the endometrial tissue, and also an increase of haptoglobin, an acute phase protein induced by inflammation process, in the blood.

Our hypothesis that pregnant animals would present a reduced expression of pro-inflammatory cytokines in circulating immune cells in comparison to non-pregnant animals was not supported. Rashid et al. (2018) reported a decreased expression of *IL1-β* and *TNF-α*, and an increase of *IL-10* in PBMC cultured and treated with uterine flushing collected from multiple embryos, on day 7 of pregnancy. Unexpectedly, and in the opposite direction, the abundance of *TNF-α* and *IL1-β* were upregulated and the abundance of *IL-10* was not affected in the PBMC on day 21 of pregnancy in the present study. In addition, significant positive correlations between *TNF-α* and *IL1-β* (r = 0.82) and between both cytokines with *ISG15* and *OAS1* (r > 0.45) were observed. Interestingly, *IL-10* an anti-inflammatory, presented significant positive correlations with all pro-inflammatory cytokines (*TNF-α, r= 0.86; IL1-β, r= 0.85 and IFN-γ,* r = 0.55). Similarly, with *TNF-α* and *IL1-β,* significant positive correlations between *IL-10* with *ISG15* and *OAS1* (r > 0.45) were observed. Recent results from Mohapatra et al. (2020) in crossbred dairy cows, indicated a greater abundance of *IFN-γ*, *TNF-α* and *IL-10* expression in PBMC of pregnant cows compared to non-pregnant on days 10 and 18 of pregnancy, but it is followed by reduced expression of both pro-inflammatory cytokines and increased of *IL-10* on day 36 of pregnancy.

Therefore, the previously described immunomodulatory effect of IFN-t toward an anti-inflammatory response, may be intensified during early phases of pregnancy related to immune tolerance to the embryo up its hatching (Rashid et al., 2018). The novel results of expression of pro-inflammatory cytokines in PBMC on the third week of pregnancy indicate that those cytokines are important factors for pregnancy establishment after the semi-allogenic embryo starts to express a major histocompatibility complex. Thus, our running hypothesis, is that after a period to recognize the semi-allogenic embryo, the adaptative maternal immune system is modulated enhancing the cascade of pro-inflammatory cytokines (Th1-response) for the establishment of pregnancy.

The accuracy of most of the early pregnancy predictors herein evaluated was reduced when SCE was diagnosed at the beginning of the TAI protocol. That is, the ROC curve analyses indicated that the early pregnancy predictors, except *ISG15*, were not effective in animals with SCE for diagnosing pregnancy on D21 after TAI. The abundance of *ISG15* proved to be a highly accurate method to predict pregnancy, independently of SCE presence. Dalmaso de Melo et al. (2020) using beef cows and heifers, indicate that the abundance of *ISG15*, *OAS1* as well as, CL blood perfusion, using Doppler-US, were good predictors of pregnancy on day 20 after TAI. The *ISG15* and *OAS1* are classic ISGs and the gene expression of these targets in PBMC is widely used as early pregnancy diagnosis in ruminants. Interestingly, in the present study, the CL blood perfusion, using the Doppler-US method was a good predictor only when applied in healthy animals, reaching 85% accuracy. The lower accuracy of CL blood perfusion, as early pregnancy predictors, when applied in SCE cows, could be associated with embryonic loss after the maternal recognition period, in those cows. However, in *Experiment 2*, there is no evidence, that cows with SCE had more embryonic loss after the maternal recognition period. Similarly, the *OAS1* abundance tended to accurately predict the pregnancy only in healthy animals. A previous report (Parent et al., 2002) indicated that animals diagnosed with SCE had a dysregulation of PGE_2_ synthases in the bovine endometrium, reducing the secretion of PGE_2_, which has a crucial role in the CL formation. Also, it is known, that the *OAS1* gene plays a crucial role in CL survival (Schmitt et al., 1993). Thus, the lower accuracy of CL blood perfusion, using Doppler-US, in animals with SCE at the beginning of the TAI protocol, could be related to changes in PGE_2_ concentrations or further factors involved in reduction of CL blood perfusion. However, one limitation of the present study to determine the interaction of pregnancy status and SCE presence is the reduced number of pregnant cows in the SCE class (n=4) and further investigations are required to understand the effect of SCE on other early pregnancy predictors and how the anti- and pro-inflammatory cytokines are modulated throughout the first three weeks of pregnancy.

## Conclusions

In conclusion, gene expression of *ISG15*, *MX1*, *IFI6*, *IFN-γ,* and *TNF-α* in PBMC are not modulated in the presence of SCE occurrence or at different estrous cycle phases in dairy cows during the postpartum period; however, *OAS1* and *IL1-β* are upregulated at diestrus, regardless SCE presence. The *MX1* and *IFI6* are not effective ISGs for use as early pregnancy markers in dairy cows with or without SCE at the beginning of the TAI protocol, as their expression is not powerfully stimulated in pregnant animals. On the other hand, *ISG15* shows to be the most efficient marker in PBMC for predicting pregnancy at 21 days post-TAI, regardless of the occurrence of SCE. Also, the efficacy of *OAS1* and Doppler-US as pregnancy predictors was reduced by SCE occurrence. Finally, pregnant animals have a greater expression of *IL1-β* and *TNF-α* on day 21 after TAI, which could indicate a shift in the maternal immune response to the conceptus toward to a Th1-response in the third week of pregnancy.
